# REM sleep is associated with white matter integrity in cognitively healthy, older adults

**DOI:** 10.1371/journal.pone.0235395

**Published:** 2020-07-09

**Authors:** Marie Altendahl, Devyn L. Cotter, Adam M. Staffaroni, Amy Wolf, Paige Mumford, Yann Cobigo, Kaitlin Casaletto, Fanny Elahi, Leslie Ruoff, Samirah Javed, Brianne M. Bettcher, Emily Fox, Michelle You, Rowan Saloner, Thomas C. Neylan, Joel H. Kramer, Christine M. Walsh

**Affiliations:** 1 Memory & Aging Center, Department of Neurology, Weill Institute for Neurosciences, University of California, San Francisco, California, United States of America; 2 San Francisco VA Medical Center, Stress & Health Research Program, Department of Mental Health, San Francisco, California, United States of America; 3 Department of Psychiatry, University of California, San Francisco, California, United States of America; 4 Rocky Mountain Alzheimer’s Disease Center, Departments of Neurosurgery and Neurology, University of Colorado Anschutz Medical Campus, Aurora, Colorado, United States of America; University of Pécs Medical School, HUNGARY

## Abstract

There is increasing awareness that self-reported sleep abnormalities are negatively associated with brain structure and function in older adults. Less is known, however, about how objectively measured sleep associates with brain structure. We objectively measured at-home sleep to investigate how sleep architecture and sleep quality related to white matter microstructure in older adults. 43 cognitively normal, older adults underwent diffusion tensor imaging (DTI) and a sleep assessment within a six-month period. Participants completed the PSQI, a subjective measure of sleep quality, and used an at-home sleep recorder (Zeo, Inc.) to measure total sleep time (TST), sleep efficiency (SE), and percent time in light sleep (LS), deep sleep (DS), and REM sleep (RS). Multiple regressions predicted fractional anisotropy (FA) and mean diffusivity (MD) of the corpus callosum as a function of total PSQI score, TST, SE, and percent of time spent in each sleep stage, controlling for age and sex. Greater percent time spent in RS was significantly associated with higher FA (β = 0.41, *p* = 0.007) and lower MD (β = -0.30, *p* = 0.03). Total PSQI score, TST, SE, and time spent in LS or DS were not significantly associated with FA or MD (p>0.13). Percent time spent in REM sleep, but not quantity of light and deep sleep or subjective/objective measures of sleep quality, positively predicted white matter microstructure integrity. Our results highlight an important link between REM sleep and brain health that has the potential to improve sleep interventions in the elderly.

## 1. Introduction

The prevalence of neurodegenerative diseases is increasing as the world’s population continues to age [1]. The number of older adults living with age-related neurodegenerative diseases is predicted to increase three-fold over the next 40 years [2]. Modifiable lifestyle factors, such as sleep, may be low-cost, highly scalable approaches to delay the onset of or even prevent age-related cognitive decline.

More than half of the older adult population experiences chronic sleep disturbances including increased sleep fragmentation, difficulty falling asleep, and decreased total sleep duration [1,3,4]. Moreover, older adults spend less time in rapid eye movement (REM) and deep sleep, more time in light sleep, and have shorter and fewer sleep cycles than younger adults [4–6]. These age-related changes in sleep and sleep architecture are hypothesized to enhance the risk of cognitive decline later in life [4,7–12]. In a prospective cohort study consisting of 1,245 cognitively intact older women, lower sleep efficiency increased the chance of developing mild cognitive impairment or dementia by 150 percent [1]. Furthermore, studies have shown that adults with a shorter duration of REM and deep sleep had poorer cognitive and memory performance the next day [10,13], and those with less REM sleep showed steeper longitudinal declines in global cognition [14]. Although the association between sleep and cognitive decline is bidirectional, the above studies indicate that poor sleep may significantly contribute to the onset of age-related neurological dysfunction.

Sleep also appears to be directly linked to age-related changes in brain structure. In functionally intact, older adults, self-reported poorer sleep quality and shorter sleep duration on the Pittsburg Sleep Quality Index (PSQI) are associated with greater reduction in total cerebral gray matter, ventricular expansion, as well as reduced hippocampal and thalamic volume on structural T1 imaging [9,15]. Additionally, fragmentation of sleep-wake rhythms has been shown to account for more than 19 percent of the variance in medial temporal lobe volumes [16]. These studies show that age-related changes in sleep may affect brain structure volumes, including regions that are important for memory.

Although many studies have reported on the relationship between sleep and grey matter, less is known about sleep’s relationship with white matter in older adults. White matter is frequently injured in sleep disorders such as obstructive sleep apnea or REM sleep behavior disorder [17,18], and reduced white matter integrity can result in slower processing speeds and declined executive function [19]. Over the past five years, studies have begun investigating the relationship between white matter microstructure assessed by diffusion tensor imaging (DTI) and subjective sleep quality measures [3,20]. In a study by Sexton et al. which followed 448 community-dwelling older adults, individuals reporting poorer current sleep quality measured by the global PSQI score demonstrated reduced global FA and increased global axial diffusivity and radial diffusivity, indicating poorer white matter microstructure integrity [3]. As one of the first imaging studies to investigate the association between sleep and white matter microstructure in community dwelling older adults, the results indicate that improving sleep quality may help to maintain white matter microstructure in aging.

While the findings reported by Sexton et al. are compelling, current sleep research has been limited by the sole use of subjective, or self-reported, sleep measures such as the PSQI or sleep diaries. Poor to moderate correlations have been measured between self-reported and objective sleep measures [21–23]. In a community-based study of 969 older adults, 34% of participants under- or over-estimated their sleep duration by more than one hour of their total sleep duration as measured by actigraphy [21]. The discrepancy between self-reported sleep duration and objective sleep duration highlights the potential bias of subjective sleep measures such as the PSQI, which incorporates self-reported sleep duration into the global PSQI score. Sleep studies reporting on associations between white matter and subjective sleep measures may be influenced by participants’ inaccurately estimating their sleep duration. To further elucidate the relationship between sleep and brain white matter metrics, it is necessary to utilize objective and reliable sleep measures.

Objective sleep measures quantify participants’ overall sleep quality (i.e. total sleep time, wake time after sleep onset, and sleep efficiency) while providing nightly information on sleep architecture and fragmentation [24]. Polysomnography (PSG), actigraphy, and at-home electroencephalographic (EEG) devices are common methods for objectively measuring sleep. Unlike PSG and actigraphy, at-home EEG sleep devices allow researchers the unique opportunity to comprehensively measure typical, habitual sleep over a series of nights under ecological validity [25]. Using an at-home, simplified EEG device, we investigated how sleep architecture relates to white matter microstructure in cognitively healthy, older adults. Given previous research showing the importance of sleep quality and duration of deep sleep and REM sleep on cognition, we hypothesized that greater sleep efficiency, total sleep time, and longer time spent in deep sleep and REM sleep would be associated with higher white matter microstructure integrity.

## 2. Material and methods

### 2.1. Participants

43 community-dwelling older adults were recruited from the longitudinal Hillblom Healthy Aging Network at the University of California, San Francisco (UCSF) Memory and Aging Center. Study exclusion criteria followed protocols described by Staffaroni et al. [26]. Exclusion from the study included diagnosis of memory impairment (dementia or MCI) or other neurological and psychiatric disorders that may impact sleep and cognition (e.g. Parkinson’s disease, epilepsy, bipolar disorder, schizophrenia). Individuals with substance use disorders or serious medical conditions such as cancer, were excluded from the study. Participants who completed an at-home sleep study, had an MRI scan within 6 months of their sleep study (either before or after), and were determined to be clinically normal by a formal committee comprised of a neurologist and board-certified neuropsychologist were included in the study. Each participant provided written, informed consent. The consent form and study protocol were approved by the UCSF Committee on Human Research.

### 2.2. General research visit

All participants completed a general research visit through ongoing observational studies in which cognitively healthy older adults participate in at the UCSF Memory and Aging Center (Hillblom Healthy Aging Network and Mechanisms of Executive Decline studies). These visits included neuropsychological testing, neurological examination, and Clinical Dementia Rating (CDR, completed via interview with study partner). A “clinically normal” diagnosis was assigned by a formal committee if at least 2 of the following criteria were met: no cognitive concerns during the neurological examination, a participant performed within expected ranges for their age and education on neuropsychological assessments, and the study informant / partner did not raise any concerns over the participant’s cognition during the CDR interview. Blood samples were processed to obtain measures of cholesterol levels (total cholesterol:high-density lipoprotein (HDL) ratio), HOMA-IR, and Apolipoprotein E (ApoE) genotype. In addition to a full medical history and general neurological examination, the neurologists measured participants’ weight, heart rate, and systolic blood pressure. Due to collection procedures, weight, heart rate, and blood pressure were not measured on all participants. A diagnosis of hyperlipidemia (HLD) was determined by chart review (self-reported diagnosis of HLD and/or cholesterol-lowering medications listed) and by cholesterol:HDL value (>3.5).

### 2.3. Lifestyle questionnaires

To assess risk of sleep apnea, participants completed the Berlin Sleep Apnea Questionnaire (BQ) [27]. The BQ is a 10-item questionnaire that classifies participants as “high risk” or “low risk” for sleep apnea. Participants completed the PSQI to better understand subjective sleep quality [28]. The PSQI is a nine-item assessment that asks participants about their sleep habits over the last month. To assess physical activity, participants completed the Physical Activity Scale for the Elderly (PASE) [29]. The PASE is an 11-item questionnaire that asks participants about their leisure time, and household and work-related activity levels. Participants reported all medications they took concurrently with the sleep study.

### 2.4. At-home sleep assessment

Participants were asked to wear a wireless sleep-monitoring device (Zeo, Inc.) in their habitual sleeping environment for up to 10 nights. Participants were instructed to place the headband on only when they were about to start trying to fall asleep. They were also instructed to keep the headband on throughout the night, and only remove it when they were ready to get up to start their day. The headband is lightweight with three dry electrodes at approximately Fp1, Fpz and Fp2. Signals are emitted from the headband to a small bedside device, which processes the electrophysiological signals in 30 second epochs using a proprietary neural network (Zeo, Inc.) to assess latency to sleep onset, duration of wake after sleep onset, light sleep (stages 1 and 2 Non-REM sleep), deep sleep (stage 3 Non-REM sleep) and REM sleep. Previous papers have shown relatively high levels of agreement between REM sleep identified by the Zeo autoscoring algorithm versus polysomnography scorers [30,31].

The first night of data collection was removed from the dataset to account for habituation to wearing the headband device. To be included in analyses, participants needed at least three additional nights of usable data, where the amount of unscored data was less than 45 minutes across the night. The unscored data may have included periods when the participant went to the restroom where signal transmission to the device may have been lost.

Light sleep, deep sleep and REM sleep were analyzed as a percent of total sleep. Sleep efficiency was calculated as the percentage of time spent asleep divided by the total time in bed trying to sleep.

### 2.5. MRI acquisition

All participants completed an MRI scan within 6 months of their sleep study (either before or after). Participants were scanned at the UCSF Neuroscience Imaging Center on a Siemens Trio 3T scanner equipped with a 64-channel head coil. Volumetric MPRAGE sequences were used to acquire T1-weighted images of the entire brain (coronal slice orientation; slice thickness = 1.0 mm; in-plane resolution = 1.0 × 1.0 mm; matrix = 240 × 256; TR = 2,300 ms; TE = 3 ms; TI = 900 ms; flip angle = 9°). Diffusion-weighted images were acquired using a single-short spin-echo sequence with the following parameters: repetition time = 5300 ms; echo time = 88 ms; inversion time = 2500 ms; flip angle = 90; field of view = 256*256 mm; 2 diffusion values of b = 0 and 1000 s/mm; 12 diffusion directions; 4 repeats; 40 slices; matrix size = 128*128;voxel size = 2 mm*2 mm; slice thickness = 3 mm; and generalized auto-calibrating partial parallel acquisition = 2.

### 2.6. DTI processing

DTI processing began with denoising [32]. The b = 0 image was co-registered with the diffusion direction images, followed by gradient direction, eddy current and distortion correction using FSL [33,34]. Diffusion tensors were calculated using a non-linear least-squares algorithm in Dipy [35]. Registration of diffusion data was accomplished through the DTI-TK software package (http://dti-tk.sourceforge.net) based on previously published methods [36]. DTI-TK implements a tensor-based registration paradigm, maximizing the alignment of white matter structures and minimizing interpolation of DTI images. An inter-subject template was created through iterative linear and non-linear registration of diffusion tensor images. Diffusion tensor images in the group space were diagonalized into eigenvectors from which FA, MD, AD, and RD maps were calculated. FA, MD, AD, and RD maps were not skeletonized, instead the mean of each metric was taken across all voxels in each tract ROI as a whole [26,37]. Global FA, MD, AD, and RD were extracted from scalar images masked with whole brain white matter by averaging across all white matter voxels.

The corpus callosum (CC) region of interest (ROI) was extracted from the ICBM-DTI-81 white matter labels and tract atlas [38]. CC FA, MD, AD, and RD values were calculated by averaging the FA, MD, AD, or RD values of three sub-regions of the CC (genu, body, and splenium). The CC, a large white matter tract in the brain, was used as an outcome measure because this region is susceptible to injury during neurodegenerative processes, white matter microstructure is reliably measured in this region, and previous sleep studies have found significant associations between sleep quality and DTI measures of the CC [3,39–45]. We conducted sensitivity analyses investigating the relationship between REM sleep and global FA/MD, covarying for age and sex, and the same pattern of results were observed (global FA: β = 0.35, *p* = 0.04, global MD: β = -0.38, *p* = 0.01). Full models analyzing the relationship between REM sleep and global FA/MD are found in S1 Table.

### 2.7. Statistical analyses

All statistical analyses were conducted using Stata Statistical Software (StataCorp. 2017. Stata Statistical Software: Release 15. College Station, TX: StataCorp LLC). Participants with less than three nights of sleep data were excluded from the analyses. To look for outliers in the sleep data, DTI data, and vascular risk measures, the range of values were assessed to ensure all values were biologically possible. Next, datapoints were visualized with histograms and outliers were identified as datapoints over 3 standard deviations from the mean. Based on this method, one outlier was identified. On the PSQI measure, one participant had a score of 17 (0.3 above the 3 standard deviation cut-off). As the PSQI represents a total score from a self-reported survey where 17 is a reasonable response, it was determined appropriate to retain this score in our analyses. No other outliers were found. Thus, all available data points were included in analyses. Due to study protocols, some participants had missing data. Participants with missing data were only excluded from the analysis directly affected by the missing data point and retained in all other analyses.

To understand the relationship between sleep and DTI measures, we performed multiple regressions controlling for age and sex (Model 1). For Model 1, separate regressions were conducted with each of the sleep metrics (PSQI (continuous measure), sleep efficiency, total sleep time, and percent time spent in light sleep, deep sleep, and REM sleep) individually as the predictor, and with the dependent variables being FA, MD, AD, or RD of the CC, as well as global FA or MD in a separate sensitivity analysis. For any of these relationships that were statistically significant, we fitted two additional regression models (Model 2 and Model 3). For Model 2, we considered several potential vascular health confounds including HOMA-IR, evidence of hyperlipidemia, systolic blood pressure, physical activity (PASE), and sleep apnea risk as measured by the BQ. To ensure that we accounted for potential collinearity of these measures, we assessed the variable inflation factor (VIF) for all covariates under consideration. HOMA-IR and hyperlipidemia were highly collinear (VIFs>8.7). As hyperlipidemia is used more often in clinical contexts, HOMA-IR was removed as a covariate. After this step, all VIFs were below 2, which, based on Craney et al. [46], enabled us to confidently proceed with our analyses. In Model 3, we controlled for ApoE gene status, in addition to age and sex, to determine if our results were independent of the ε4 genotype, which is associated with sleep disturbances [47,48]. We also performed follow-up analyses to determine if sleep-modifying medications contributed to the significant relationship between CC FA/MD and REM sleep.

## 3. Results

### 3.1. Demographics

Participants had a mean age of 75 ± 4.51 years (66–84 years), mean education of 18 ± 2.1 years (13–20 years), and 27 participants identified as female. All participants were cognitively normal with a mean mini-mental state examination (MMSE) of 29 ± 1.19 (26–30) and CDR box score of 0. A total of 7 participants took sleep modifying medications (sleep enhancement medication, anxiolytics, and/or antidepressants). Additional data describing DTI values and vascular risk factors are presented in Table 1.

**Table 1:** Diffusion tensor imaging and vascular risk factor data.

	N	Mean	Std. Dev.	Range
*Corpus Callosum DTI*				
Fractional Anisotropy (FA) of Corpus Callosum (CC)	43	0.54	0.04	0.46–0.64
Mean Diffusivity (MD) of Corpus Callosum (CC)	43	0.75	0.06	0.63–0.87
*Vascular Risk Factors*				
Systolic Blood Pressure	35	134.8	19.1	104–186
PASE	32	116.6	38.4	47–233
Diagnosis of Hyperlipidemia (HLD) (N, % with HLD)	43, 56%	**-**	**-**	**-**
Berlin Questionnaire (N, % at High Risk for OSA)	41, 14.6%	**-**	**-**	**-**

DTI and vascular risk factor data for subject cohort of 43 cognitively normal, older adults. All subjects participated in an MRI scan on a Siemens Trio 3T scanner, fasting blood draw, and comprehensive neurological assessment. DTI = diffusion tensor imaging, FA = fractional anisotropy, MD = mean diffusivity, HDL = high-density lipoprotein, bpm = beats per minute, PASE = Physical Activity Scale for the Elderly.

### 3.2. Sleep parameters

Participants included in our study had an average PSQI of 5.31 ± 3.79 (1–17). 13 out of 42 participants with PSQI data had a PSQI score greater than 5 and were classified as “poor” sleepers. PSQI data, however, were analyzed as a continuous variable. 6 out of 41 participants were classified as “high risk” for sleep apnea based on the BQ. Across the nights, participants spent an average of 52.2% ± 8.73% (28.2–68.7%) in light sleep, 7.75% ± 4.01 (0.73–17.9%) in deep sleep, and 26.6% ± 7.79% (11.0–47.0%) in REM sleep. Additionally, participants had a mean total sleep time of 6.45 hrs ± 0.81 hrs (4.27–7.83 hrs) and mean sleep efficiency of 84.2% ± 32.3% (64.3–97.25%) (Table 2).

**Table 2:** Sleep architecture and sleep quality data.

	N	Mean	Std. Dev.	Range
Sleep efficiency (%)	43	84.2	8.15	64.3–97.3
Total sleep time (hrs)	43	6.45	0.81	4.27–7.83
Light Sleep (%)	43	52.2	8.73	28.2–68.7
Deep Sleep (%)	43	7.75	4.01	0.73–17.9
REM Sleep (%)	43	26.6	7.79	11.0–47.0
PSQI	42	5.31	3.79	1.0–17.0

Descriptive statistics for sleep architecture and sleep quality data for subject cohort of 43 cognitively normal, older adults. PSQI = Pittsburg Sleep Quality Index, REM = rapid eye movement.

### 3.3. Sleep architecture and white matter microstructure

We present associations with callosal DTI as a proxy for global white matter integrity due to its susceptibility in various neurodegenerative disease processes [49]. We performed a sensitivity analysis comparing global DTI metrics to sleep measures, which showed a similar pattern of results as CC DTI metrics and sleep measures.

**Fig 1:**
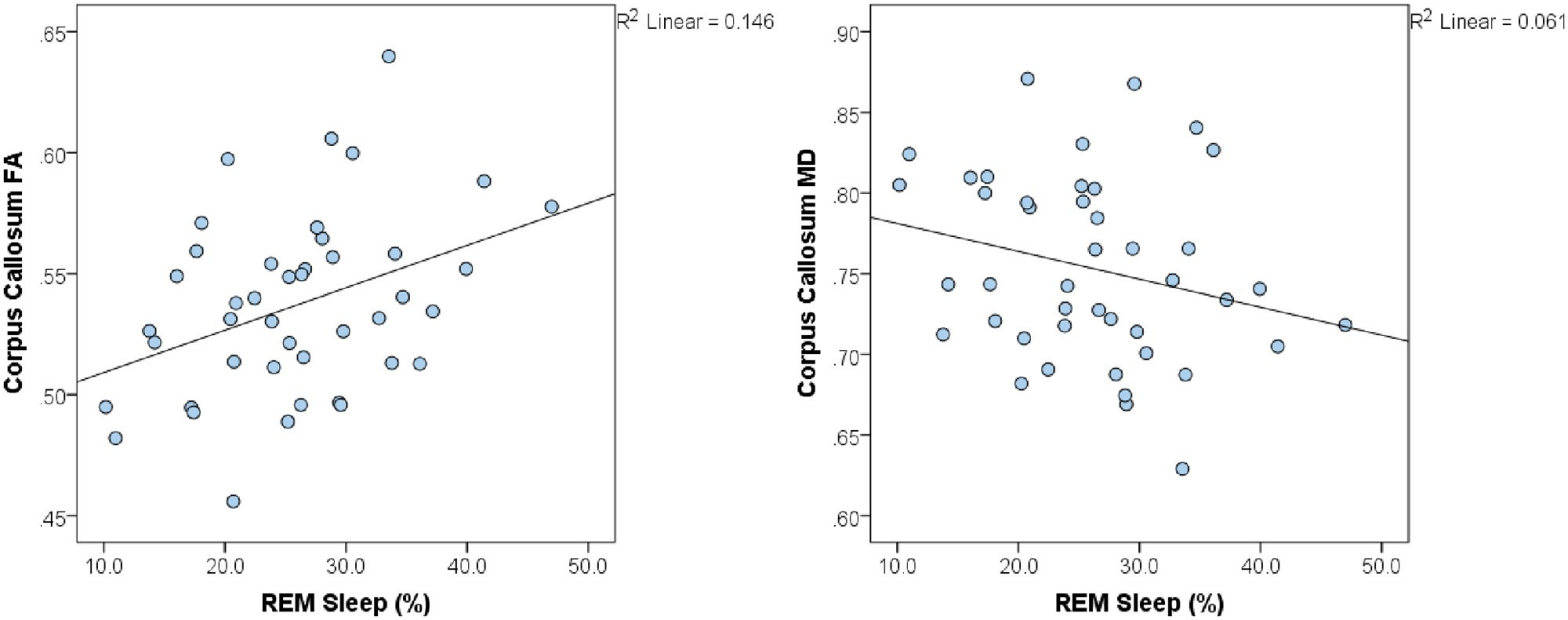
Percent of time spent in REM sleep is significantly associated with FA and MD of the corpus callosum. REM sleep percentage was significantly associated with FA and MD of the corpus callosum (FA: *p* = 0.007, MD: *p* = 0.03). Each data point represents one subject, with the solid line representing the line of best fit. REM = rapid eye movement, FA = fractional anisotropy, MD = mean diffusivity.

Adjusting for age and sex (Table 3, Model 1), the percent of time spent in REM sleep was significantly associated with CC FA (β = 0.41, *p* = 0.007) and CC MD (β = -0.30, *p* = 0.03) (Fig 1). Of note, REM sleep was also significantly associated with CC RD (β = -0.37, *p* = 0.008), but not CC AD (β = -0.15, *p* = 0.32) (Table 4, Model 1). Similar results were observed when comparing global FA (β = 0.35, *p* = 0.04), MD (β = -0.38, *p* = 0.01), AD (β = -0.24, *p* = 0.10), and RD (β = -0.25, *p* = 0.10 to percent of time spent in REM sleep (S1 Table, Model 1; S2 Table, Model 1). Percent time spent in light sleep and deep sleep did not significantly relate to the FA or MD of the corpus callosum (Table 5).

**Table 3:** Regression models of REM sleep and DTI of corpus callosum FA and MD.

	beta	*t*	95% CI	*p*	Partial η^2^	beta	*t*	95% CI	*P*	Partial η^2^
*Model 1*: *REM Sleep*	*A*. *FA of Corpus Callosum (N = 43)*, *R*^*2*^ *Adj = 0*.*233197*					*B*. *MD of Corpus Callosum (N = 43)*, *R*^*2*^ *Adj = 0*.*35023*				
Age	-0.37	-2.75	-0.005, -0.0008	0.009**	0.159	0.56	4.46	0.004, 0.010	<0.0001**	0.352
Sex	-0.13	-0.89	-0.016, 0.006	0.38	0.018	0.12	0.89	-0.009, 0.022	0.38	0.020
REM Sleep (%)	0.41	2.85	0.0006, 0.003	0.007**	0.196	-0.30	-2.29	-0.004, -0.0003	0.03*	0.133
*Model 2*: *REM Sleep and Vascular Risk*	*A*. *FA of Corpus Callosum (N = 33)*, *R*^*2*^ *Adj = 0*.*092024)*					*B*. *MD of Corpus Callosum (N = 33)*, *R*^*2*^ *Adj = 0*.*334091*				
Age	-0.40	-2.00	-0.006, 8.8e-5	0.05*	0.138	0.60	3.52	0.003, 0.012	0.0004**	0.332
Sex	0.09	0.44	-0.02, 0.03	0.66	0.008	-0.13	-0.79	-0.06, 0.025	0.0499*	0.025
Cholesterol Risk	-0.17	-0.91	-0.02, 0.007	0.37	0.032	0.13	0.84	-0.011, 0.028	0.28	0.027
Systolic Blood Pressure	-0.02	-0.13	-0.0007, 0.0006	0.89	0.001	0.09	0.56	-0.0006, 0.0011	0.99	0.013
Berlin Risk	-0.04	-0.22	-0.04, 0.03	0.82	0.002	0.12	0.83	-0.03, 0.07	0.62	0.027
PASE	-0.02	-0.15	-0.0004, 0.0003	0.88	0.001	-0.14	-0.94	-0.0007, 0.0002	0.19	0.034
REM Sleep (%)	0.47	2.24	0.0002, 0.004	0.03*	0.167	-0.40	-2.21	-0.006, -0.0002	0.037*	0.163
*Model 3*: *REM Sleep and ApoE*	*A*. *FA of Corpus Callosum (N = 42)*, *R*^*2*^ *Adj = 0*.*222068*					*B*. *MD of Corpus Callosum (N = 42)*, *R*^*2*^ *Adj = 0*.*360989*				
Age	-0.34	-2.42	-0.005, -0.0005	0.021*	0.134	0.60	4.65	0.004, 0.011	<0.0001**	0.383
Sex	-0.15	-1.06	-0.02, 0.005	0.29	0.028	0.07	0.51	-0.012, 0.02	0.45	0.007
ApoE Polymorphism	0.028	0.20	-0.011, 0.028	0.85	0.0003	0.002	0.02	-0.017, 0.017	0.72	0.0001
REM Sleep (%)	0.43	2.82	0.0006, 0.004	0.008**	0.206	-0.26	-1.87	-0.004, -0.0002	0.07	0.101

DTI = diffusion tensor imaging, REM = rapid eye movement, FA = fractional anisotropy, MD = mean diffusivity, PSQI = Pittsburg Sleep Quality Index, ApoE = Apolipoprotein E, BQ = Berlin Sleep Apnea Questionnaire, BP = blood pressure, PASE = Physical Activity Scale for the Elderly.

* = p<0.05,

** = p<0.01.

**Table 4:** Regression models of REM sleep and DTI of corpus callosum AD and RD.

	beta	*t*	95% CI	*p*	Partial η^2^	beta	*t*	95% CI	*p*	Partial η^2^
*Model 1*: *REM Sleep*	*A*. *AD of Corpus Callosum (N = 43)*, *R*^*2*^ *Adj = 0*.*219278*					*B*. *RD of Corpus Callosum (N = 43)*, *R*^*2*^ *Adj = 0*.*360796*				
Age	0.50	3.67	0.003, 0.012	0.0007**	0.275	0.53	4.29	0.003, 0.010	<0.0001**	0.329
Sex	0.07	0.45	-0.016, 0.025	0.65	0.006	0.14	1.04	-0.007, 0.023	0.30	0.027
REM Sleep (%)	-0.15	-1.00	-0.004, 0.001	0.32	0.027	-0.37	-2.81	-0.005, -0.0007	0.008**	0.19
*Model 2*: *REM Sleep and Vascular Risk*	*A*. *AD of Corpus Callosum (N = 33)*, *R*^*2*^ *Adj = 0*.*269165*					*B*. *RD of Corpus Callosum (N = 33)*, *R*^*2*^ *Adj = 0*.*299156*				
Age	0.54	3.07	0.003, 0.015	0.005**	0.274	0.57	3.31	0.003, 0.012	0.003**	0.304
Sex	-0.1	-0.57	-0.068, 0.039	0.58	0.013	-0.14	-0.83	-0.056, 0.024	0.41	0.027
Cholesterol Risk	0.07	0.43	-0.021, 0.032	0.67	0.007	0.16	0.99	-0.01, 0.03	0.33	0.038
Systolic Blood Pressure	0.11	0.65	-0.0008, 0.002	0.52	0.017	0.07	0.43	-0.0007, 0.001	0.67	0.007
Berlin Risk	0.14	0.89	-0.039, 0.099	0.38	0.031	0.11	0.66	-0.034, 0.068	0.51	0.017
PASE	-0.21	-1.29	-0.001, 0.0002	0.21	0.063	-0.09	-0.57	-0.0006, 0.0003	0.57	0.013
REM Sleep (%)	-0.25	-1.31	-0.006, 0.001	0.20	0.064	-0.47	-2.48	-0.006, -0.0005	0.02*	0.198
*Model 3*: *REM Sleep and ApoE*	*A*. *AD of Corpus Callosum (N = 42)*, *R*^*2*^ *Adj = 0*.*26721*					*B*. *RD of Corpus Callosum (N = 42)*, *R*^*2*^ *Adj = 0*.*350845*				
Age	0.59	4.26	0.005, 0.013	<0.0001**	0.34659	0.54	4.18	0.010, 0.544	0.0002**	0.331
Sex	-0.01	-0.10	-0.021, 0.019	0.92	0.00011	0.11	0.81	-0.009, 0.021	0.42	0.017
ApoE Polymorphism	0.05	0.35	-0.018, 0.025	0.72	0.00296	-0.03	-0.19	-0.019, 0.015	0.85	0.001
REM Sleep (%)	-0.08	-0.57	-0.003, 0.002	0.57	0.00878	-0.33	-2.42	-0.004, -0.0004	0.021*	0.160

DTI = diffusion tensor imaging, REM = rapid eye movement, AD = axial diffusivity, RD = radial diffusivity, PSQI = Pittsburg Sleep Quality Index, ApoE = Apolipoprotein E, BQ = Berlin Sleep Apnea Questionnaire, BP = blood pressure, PASE = Physical Activity Scale for the Elderly.

* = p<0.05,

** = p<0.01.

**Table 5:** Associations between sleep data and DTI.

	Beta	*t*	p-value
*Outcome Variable*: *CC FA*			
PSQI	-0.08	-0.49	0.63
Sleep efficiency (%)	-0.13	-0.85	0.4
Total sleep time (min)	0.11	0.73	0.47
Light Sleep (%)	-0.23	-1.56	0.13
Deep Sleep (%)	0.03	0.18	0.861
REM Sleep (%)	0.41	2.85	0.007**
*Outcome Variable*: *CC MD*			
PSQI	0.18	1.28	0.21
Sleep efficiency (%)	-0.06	-0.44	0.67
Total sleep time (min)	-0.15	-1.13	0.26
Light Sleep (%)	0.14	1.03	0.31
Deep Sleep (%)	0.02	0.12	0.91
REM Sleep (%)	-0.30	-2.29	0.027*

CC FA = corpus callosum fractional anisotropy, CC MD = corpus callosum mean diffusivity, PSQI = Pittsburg Sleep Quality Index, REM = rapid eye movement.

* = p<0.05,

** = p<0.01.

The relationships between REM sleep and CC FA/MD/RD were independent of vascular risk factors and ApoE status (Table 3, Models 2A, 2B, and 3A; Table 4, Models 2B and 3B), with the exception of the relationship between CC MD and REM sleep when controlling for ApoE (Table 3, Model 3B). CC AD was not significantly related to REM sleep when controlling for ApoE gene status or controlling for vascular risk factors (*p*’s > 0.20) (Table 4, Models 2A and 3A).

There were no group differences in percent of REM sleep between those taking sleep modifying medications and those who do not (*p* = 0.277). Further, the relationship between REM sleep and CC FA/MD remained significant after removing the 7 individuals taking sleep modifying medication (CC FA: β = 0.36, *p* = 0.046, CC MD: β = -0.35, *p* = 0.030).

### 3.4. Sleep quality and white matter microstructure

Objectively measured total sleep time (CC FA: β = 0.11, *p* = 0.47, CC MD: β = -0.152, *p* = 0.26) and sleep efficiency (CC FA: β = -0.13, *p* = 0.40; CC MD: β = -0.06, *p* = 0.67) were not significantly associated with CC FA or CC MD (Table 5). Subjective sleep quality determined by the PSQI did not significantly relate to white matter microstructure (CC FA: β = -0.09, *p* = 0.61; CC MD: β = 0.18, *p* = 0.23).

## 4. Discussion

The goal of our study was to further investigate the relationship between objective sleep measures and white matter microstructure using an at-home EEG sleep device and DTI. We aimed to evaluate how sleep efficiency, total sleep time, and time spent (percent) in light sleep, deep sleep, and REM sleep, related to white matter microstructure. Surprisingly, we found that greater time spent in REM sleep (percent) was significantly related to higher CC FA, lower CC MD values, and lower CC RD values. Our results indicate that increased duration of REM sleep relates to healthier white matter microstructure in cognitively normal older adults, independent of sleep apnea, vascular, or genetic risk factors.

### 4.1. Measures of sleep quality

Notably, total PSQI score, a subjective measure of sleep quality, did not relate to CC FA or CC MD in our cohort of cognitively normal, older adults. These results are inconsistent with previous research findings that report a significant relationship between total PSQI score and DTI in healthy older adults and individuals with insomnia [3,40]. Our cohort of healthy older adults had a mean age of 75 years and was older than the cohort in Sexton et al., which had a mean age of 69 years. Both sleep disturbances and white matter injury increase with age, and this age difference may be contributing to the differing study outcomes [3,4,7,50]. Additionally, our results may have been inconsistent with Sexton et al. because we compared total PSQI score to a mean measure of CC FA and MD, as a proxy for whole brain white matter, while they used various DTI ROIs and CC sub-regions. Finally, our cohort consisted of 43 individuals, compared to 348 in Sexton et al. Although the percentages of individuals reporting “poor” sleep quality were similar in both studies, the differing sample sizes may also contribute to our contradicting results. The PSQI may have a small effect size, and our cohort of 43 participants may not have been sufficient to find a significant result.

Our results are consistent with recent studies investigating the relationship between measures of sleep efficiency and sleep duration with brain DTI measures [3,51]. In our study, objectively measured sleep efficiency and total sleep time, both measures of sleep quality, did not significantly relate to CC FA or CC MD. Spiegelhalder et al. found a similar result in their cohort of 24 participants with insomnia. All 24 participants completed an MRI scan and two consecutive nights of PSG sleep recording. Spiegelhalder et al. found no significant relationship between objectively measured total sleep time and FA outcome measures [51]. Furthermore, a similar result was found when comparing subjectively measured sleep duration and sleep efficiency to DTI metrics. Sexton et al. derived measures of sleep duration and sleep efficiency from the PSQI on 448 healthy older adults. Sexton et al. did not find a significant relationship between self-reported sleep duration or sleep efficiency with DTI metrics. Our results support previous studies that indicate no relationship between sleep duration, sleep efficiency and brain white matter microstructure.

Although previous research indicates an association between subjective measures of sleep quality (e.g. PSQI or sleep diaries) and white matter microstructure, it is unsurprising that few studies have found similar results when objectively measuring sleep quality. Several studies have reported a high-level of disagreement between objective and subjective sleep quality measures in older adults [21–23]. Subjective sleep measures are influenced by personal characteristics such as sex, age, degree of cognitive impairment, and mood and may introduce subject bias when evaluating sleep quality [21]. The unreliability of subjective sleep measures supports the need for future sleep studies to include both subjective and objective sleep measures.

### 4.2. REM sleep and neural white matter

There are several factors that could have contributed to our finding that the percent time spent in REM sleep was associated with white matter FA and MD in cognitively normal healthy older adults. We included many of these in our analyses to determine if the finding may be specific to REM sleep itself or to other contributing factors such as vascular health, obstructive sleep apnea, and ApoE status. These are discussed below.

### 4.3. Vascular health, obstructive sleep apnea, and neural white matter health

Research has shown that white matter microstructure reduces with age and is sensitive to vascular risk modifiers such as hypertension, high body mass index (BMI), high cholesterol, and low exercise [52–54]. Notably, the relationship between the percent time spent in REM sleep and CC FA/MD remained significant after co-varying for vascular risk factors. Therefore, the specific association between the percent time in REM sleep and CC FA and CC MD was not explained by variation in vascular health in this cohort.

Furthermore, moderate to severe obstructive sleep apnea (OSA) contributes to cerebral white matter change [55]. OSA is a sleep disorder characterized by obstruction to the upper airway throughout the night [17]. During OSA, it is hypothesized that the brain enters a state of hypoxia, altering cerebral blood flow, and resulting in cerebrovascular shearing [17]. Individuals with OSA spend less time in REM sleep because OSA episodes occur more frequently and last longer during REM sleep compared to non-REM sleep [56,57]. In our cohort, 6 participants were at high risk for sleep apnea as determined by the BQ. After co-varying for sleep apnea risk and other vascular risk factors (systolic blood pressure, cholesterol risk, and exercise) the relationship between REM sleep and CC FA/MD remained significant. Further research is required to determine the role of sleep apnea in this relationship using an objective, physiologic measure of sleep apnea; however, in our current data set using the BQ, we demonstrated that self-reported OSA did not strongly impact our findings.

### 4.5. ApoE gene status and neural white matter health

ApoE-ε4 carriers are at higher risk for cognitive decline than their non-ε4 counterparts [47]. Cognitive impairment is associated with reduced REM sleep and increased slow-wave sleep disturbances [48]. Additionally, ApoE-ε4 carriers with mild cognitive impairment (MCI) are more likely to have decreased REM sleep duration than non-carriers with MCI [48]. We therefore assessed the contribution of Apo-ε4 gene status to our findings. The association between REM sleep and CC FA/MD was independent of ApoE polymorphism. It would be of interest in future studies to incorporate amyloid and tau measures to identify how they are associated with the REM sleep and white matter relationship we are describing here.

### 4.6. REM sleep and neural white matter health

The potential mechanisms underlying the relationship between REM sleep and brain white matter microstructure remain unclear mechanistically. Oligodendrocyte precursor cells (OPCs) develop oligodendrocytes, which are the cells that produce myelin. In studies of OPCs and sleep, REM sleep alone was positively correlated with OPC proliferation [58]. Though OPC proliferation decreases with age and may not be occurring in our older adult cohort, this previous study supports a link between REM sleep and the promotion of white matter structural health. In further support of this potential link, our data suggests that the relationship between FA/MD and REM sleep was being driven by RD, not AD. This result could be evidence that the DTI association with REM sleep is driven by myelin loss, rather than axonal loss, however, not all research supports the validity of drawing such inferences from the AD vs RD distinction [59,60]. Future research using more sophisticated models such as neurite orientation dispersion and density imaging (NODDI) could help elucidate the biological underpinnings of this finding.

REM sleep has also been associated with regulating the chemical and physical properties of the blood brain barrier (BBB). One study found that mice deprived of REM sleep had increased BBB permeability [61]. This increase in BBB permeability could have negative consequences on overall brain health. BBB dysfunction has been associated with increased levels of inflammation and subsequent damage to brain white matter microstructure [62–65]. These studies suggest that individuals with lower amounts of REM sleep in our cohort may be at greater risk for BBB dysfunction that contributes to downstream white matter injury.

High amplitude-theta wave bursts are characteristic of REM sleep [66], and a recent study indicated that the theta waves may have a restorative effect on white matter injury [67]. Using a rat stroke model, Segal et al. divided 18 injured rats into three treatment groups. Injured rats exposed to an electromagnetic field with the frequency of theta waves for four weeks showed greater white matter integrity on brain MRI compared to controls and had histological evidence for neuronal regeneration and plasticity [68]. This preliminary study suggests that theta waves may promote recovery of brain white matter.

### 4.7. Limitations

This study was limited by its relatively small sample size (n = 43) and lack of longitudinal data. In some cases, it was further limited with missing variables for some participants. Our results highlight an important link between REM sleep and brain health that has the potential to improve sleep interventions in the elderly; yet, our study sample was highly educated, which may limit the generalizability of our findings to more socio-economically diverse populations. Sleep quantity and quality greatly varies with age and neurodegenerative disease processes [67]. Although we were able to collect 3–10 nights of sleep data, we were unable to obtain longitudinal sleep measures to investigate how changes in sleep habits over time influence brain white matter integrity. Furthermore, our study did not assess objective measures of sleep apnea. Quantifying sleep apneic events will help elucidate the relationship between REM sleep and white matter microstructure. Finally, additional measurements of white matter injury such as inflammation, may give insight into how REM sleep and brain white matter relate mechanistically. Future studies with larger sample sizes, longitudinal sleep data, objective measures of sleep apnea, and molecular assessments of brain injury, such as inflammation, are needed to further investigate the relationship between REM sleep and brain white matter.

## 5. Conclusions

Overall, our study found that subjective (total PSQI score) and objective measures (sleep efficiency and total sleep time) of sleep quality did not relate to CC FA/MD. Our results, however, do indicate that duration of REM sleep may be a greater contributor to brain white matter integrity than overall sleep quality. It is imperative that future research objectively measures sleep architecture in addition to sleep quality to elucidate the relationship between sleep and brain white matter.

## Supporting information

S1 Table (Global FA and Global MD). (DOCX)

S2 Table (Global AD and Global RD). (DOCX)

S3 Table (GMV). (DOCX)

## Abbreviations

ApoE: Apolipoprotein
BBB: Blood Brain Barrier
BMI: Body Mass Index
BQ: Berlin Sleep Apnea Questionnaire
CC: Corpus Callosum
CDR: Clinical Dementia Rating
DS: Deep Sleep
DTI: Diffusion Tensor Imaging
EEG: Electroencephalography
FA: Fractional Anisotropy
HDL: High Density Lipoprotein
HOMA-IR: Homeostatic Model Assessment for Insulin Resistance
LS: Light Sleep
MCI: Mild Cognitive Impairment
MD: Mean Diffusivity
MMSE: Mini-Mental State Examination
OSA: Obstructive Sleep Apnea
PSG: Polysomnography
PSQI: Pittsburg Sleep Quality Index
REM: Rapid Eye Movement
ROI: Region of Interest
RS: REM Sleep
SE: Sleep Efficiency
TST: Total Sleep Time
WM: White Matter
