# Supplementary material for: REM sleep is associated with white matter integrity in cognitively healthy, older adults
Source: PLoS One. 2020 Jul 9;15(7):e0235395. doi: 10.1371/journal.pone.0235395 (PMC7347149; doi:10.1371/journal.pone.0235395)
Supplement: S1 Table — (DOCX) [file pone.0235395.s001.docx]

Table 1 (Global FA and Global MD):

|  | **Beta** | ***t*** | **95% CI** | ***p*** | **Partial η^2^** | **beta** | ***t*** | **95% CI** | ***p*** | **Partial η^2^** |
| --- | --- | --- | --- | --- | --- | --- | --- | --- | --- | --- |
| *Model 1: REM Sleep* | *A. Global FA (N=42), R^2^ Adj = 0.114896* | | | | | *B. Global MD (N=42), R^2^ Adj = 0.286115* | | | | |
| Age | -0.26 | -1.75 | -0.001, 0.0001 | 0.09 | 0.06043 | 0.44 | 3.32 | 0.0009, 0.004 | 0.002** | 0.2074 |
| Sex | -0.13 | -0.79 | -0.006, 0.002 | 0.43 | 0.01433 | 0.07 | 0.49 | -0.005, 0.008 | 0.63 | 0.00523 |
| REM Sleep (%) | 0.35 | 2.14 | 3.03e-5, 0.001 | 0.04* | 0.10272 | -0.38 | -2.64 | -0.002, -0.0002 | 0.01* | 0.12425 |
| *Model 2: REM Sleep and Vascular Risk* | *A. Global FA (N=32), R^2^ Adj = -0.01851* | | | | | *B. Global MD (N=32), R^2^ Adj = 0.246665* | | | | |
| Age | -0.25 | -1.19 | -0.002, 0.0005 | 0.24 | 0.0558 | 0.50 | 2.73 | 0.0006, 0.004 | 0.01** | 0.23665 |
| Sex | 0.08 | 0.35 | -0.009, 0.013 | 0.72 | 0.00513 | -0.07 | -0.33 | -0.021, 0.015 | 0.74 | 0.00457 |
| Cholesterol Risk | -0.01 | -0.05 | -0.005, 0.005 | 0.95 | 0.00011 | 0.05 | 0.31 | -0.007, 0.009 | 0.76 | 0.00391 |
| Systolic Blood Pressure | -0.12 | -0.58 | -0.0003, 0.0002 | 0.56 | 0.01371 | 0.03 | 0.19 | -0.0004, 0.0004 | 0.84 | 0.00155 |
| Berlin Risk | -0.07 | -0.35 | -0.014, 0.010 | 0.73 | 0.00502 | 0.03 | 0.16 | -0.019, 0.022 | 0.87 | 0.00105 |
| PASE | 0.05 | 0.27 | -0.0001, 0.0001 | 0.78 | 0.00304 | -0.22 | -1.35 | -0.0003, 0.00006 | 0.19 | 0.07033 |
| REM Sleep (%) | 0.36 | 1.48 | -0.0002, 0.001 | 0.15 | 0.08405 | -0.49 | -2.30 | -0.002, -0.0001 | 0.03* | 0.18118 |
| *Model 3: REM Sleep and ApoE* | *A. Global FA (N=41), R^2^ Adj = 0.125772* | | | | | *B. Global MD (N=41), R^2^ Adj = 0.299854* | | | | |
| Age | -0.28 | -1.84 | -0.002, 7.85e-5 | 0.07 | 0.06926 | 0.49 | 3.57 | 0.001, 0.004 | 0.001** | 0.23573 |
| Sex | -0.15 | 0.87 | -0.006, 0.002 | 0.39 | 0.01829 | 0.05 | 0.34 | -0.006, 0.008 | 0.74 | 0.0023 |
| ApoE Polymorphism | -0.19 | -1.20 | -0.007, 0.002 | 0.24 | 0.03202 | 0.17 | 1.26 | -0.003, 0.012 | 0.22 | 0.02552 |
| REM Sleep (%) | 0.41 | 2.38 | 0.0001, 0.0012 | 0.02* | 0.12408 | -0.41 | -2.65 | -0.002, -0.0002 | 0.01* | 0.12086 |
