# Supplementary material for: REM sleep is associated with white matter integrity in cognitively healthy, older adults
Source: PLoS One. 2020 Jul 9;15(7):e0235395. doi: 10.1371/journal.pone.0235395 (PMC7347149; doi:10.1371/journal.pone.0235395)
Supplement: S2 Table — (DOCX) [file pone.0235395.s002.docx]

Table 2 (Global AD and Global RD):

|  | **beta** | ***t*** | **95% CI** | ***p*** | **Partial η^2^** | **beta** | ***t*** | **95% CI** | ***p*** | **Partial η^2^** |
| --- | --- | --- | --- | --- | --- | --- | --- | --- | --- | --- |
| *Model 1: REM Sleep* | *A. Global AD (N=43), R^2^ Adj = 0.272898* | | | | | *B. Global RD (N=43), R^2^ Adj = 0.223194* | | | | |
| Age | 0.52 | 3.90 | 0.001, 0.004 | 0.0004** | 0.26554 | 0.47 | 3.46 | 0.001, 0.004 | 0.001** | 0.21653 |
| Sex | -0.08 | -0.56 | -0.009, 0.005 | 0.58 | 0.00741 | -0.03 | -0.21 | -0.008, 0.006 | 0.84 | 0.00131 |
| REM Sleep (%) | -0.24 | -1.71 | -0.002, 0.0001 | 0.10 | 0.04716 | -0.25 | -1.70 | -0.002, 0.0001 | 0.10 | 0.0566 |
| *Model 2: REM Sleep and Vascular Risk* | *A. Global AD (N=33), R^2^ Adj = 0.274096* | | | | | *B. Global RD (N=33), R^2^ Adj = 0.200644* | | | | |
| Age | 0.59 | 3.36 | 0.001, 0.005 | 0.003** | 0.31057 | 0.52 | 2.84 | 0.0007, 0.004 | 0.009** | 0.24451 |
| Sex | 0.14 | 0.81 | -0.011, 0.025 | 0.43 | 0.02537 | 0.11 | 0.61 | -0.012, 0.022 | 0.55 | 0.01448 |
| Cholesterol Risk | 0.14 | 0.82 | -0.005, 0.012 | 0.42 | 0.0261 | 0.12 | 0.67 | -0.006, 0.011 | 0.51 | 0.01742 |
| Systolic Blood Pressure | 0.11 | 0.64 | -0.0003, 0.0005 | 0.53 | 0.01604 | 0.17 | 0.99 | -0.0002, 0.0006 | 0.33 | 0.0376 |
| Berlin Risk | 0.01 | 0.07 | -0.022, 0.024 | 0.94 | 0.00022 | 0.04 | 0.25 | -0.020, 0.025 | 0.81 | 0.00247 |
| PASE | -0.22 | -1.36 | -0.0004, 7.41e-5 | 0.19 | 0.06851 | -0.14 | -0.82 | -0.0003, 0.0001 | 0.42 | 0.02607 |
| REM Sleep (%) | -0.29 | -1.50 | -0.002, 0.0003 | 0.14 | 0.0822 | -0.26 | -1.33 | -0.002, 0.0004 | 0.20 | 0.06622 |
| *Model 3: REM Sleep and ApoE* | *A. Global AD (N=42), R^2^ Adj = 0.284751* | | | | | *B. Global RD (N=42), R^2^ Adj = 0.225859* | | | | |
| Age | 0.56 | 4.13 | 0.002, 0.005 | 0.0002** | 0.29349 | 0.51 | 3.61 | 0.001, 0.004 | 0.0009** | 0.23825 |
| Sex | -0.12 | -0.84 | -0.011, 0.004 | 0.41 | 0.01699 | -0.05 | -0.38 | -0.012, 0.004 | 0.71 | 0.00392 |
| ApoE Polymorphism | 0.10 | 0.73 | -0.006, 0.011 | 0.47 | 0.00654 | 0.12 | 0.89 | -0.009, 0.006 | 0.38 | 0.01465 |
| REM Sleep (%) | -0.22 | -1.54 | -0.002, 0.0002 | 0.13 | 0.03506 | -0.25 | -1.66 | -0.002, 0.0002 | 0.11 | 0.05261 |
