# Supplementary material for: REM sleep is associated with white matter integrity in cognitively healthy, older adults
Source: PLoS One. 2020 Jul 9;15(7):e0235395. doi: 10.1371/journal.pone.0235395 (PMC7347149; doi:10.1371/journal.pone.0235395)
Supplement: S3 Table — (DOCX) [file pone.0235395.s003.docx]

Table 3 (GMV):

| *REM Sleep and GMV (N=36),* *R^2^ Adj = 0.562164* | | | | | |
| --- | --- | --- | --- | --- | --- |
|  | **beta** | ***t*** | **95% CI** | ***p*** | **Partial η^2^** |
| Age | -0.20 | -1.67 | -0.005, 0.0005 | 0.11 | 0.13774 |
| Sex | 0.21 | 0.97 | -0.01, 0.03 | 0.33 | 0.00602 |
| TIV | 0.66 | 3.15 | 0.08, 0.37 | 0.004** | 0.28314 |
| REM Sleep (%) | -0.08 | -0.66 | -0.002, 0.001 | 0.51 | 0.00032 |
